# “Trained Immunity” from *Mycobacterium* spp. exposure (BCG vaccination and environmental) may have an impact on the incidence of early childhood leukemia

**DOI:** 10.3389/fimmu.2023.1193859

**Published:** 2023-05-24

**Authors:** Samer Singh, Dhiraj Kishore, Rakesh K. Singh

**Affiliations:** ^1^ Centre of Experimental Medicine & Surgery, Institute of Medical Sciences, Banaras Hindu University, Varanasi, India; ^2^ Department of General Medicine, Institute of Medical Sciences, Banaras Hindu University, Varanasi, India; ^3^ Department of Biochemistry, Institute of Science, Banaras Hindu University, Varanasi, India

**Keywords:** *Mycobacterium* spp., childhood leukemia, childhood vaccinations, BCG vaccine, trained immunity, MCV2, PCV3, DTP3

## Abstract

Preventive variables for childhood leukemia incidence (LI) remain unknown. Past assertions that childhood vaccinations, especially BCG, may be potentially protective have remained disputed for over five decades because of the lack of a unifying framework to explain variable outcomes in different studies. An examination of the early childhood LI for 2020 in European Region countries with supposedly similar underlying confounders but differential childhood vaccination coverage displays negative covariation with prevailing *Mycobacterium* spp. exposure in BCG-vaccinated children. The childhood LI in 0–4-year-old populations with >90% childhood BCG vaccination coverage is found to be strongly but negatively correlated with prevailing tuberculin immunoreactivity [*r(24)*: -0.7868, *p-value*: < 0.0001]. No such correlation existed for the LI in 0–4-year-old populations without BCG vaccinations, though weak associations are hinted at by the available data for MCV2, PCV3, and DTP3 vaccinations. We hypothesize that early childhood BCG vaccination “priming” and subsequent “trained immunity” augmentation by “natural” boosting from *Mycobacterium* spp. exposure play a preventive and protective role in childhood LI. The non-consideration of prevailing “trained immunity” could have been a cause behind the conflicting outcomes in past studies. Exploratory studies, preferably performed in high-burden countries and controlling for the trained-immunity correlate and other potential confounders, would be warranted in order to establish a role for BCG vaccination and early-life immune training (or lack thereof) in childhood LI and help put the current controversy to rest.

## Introduction

Many excellent observational studies (1–4) and expositions (5–9) in the past have suggested a role for early Bacille Calmette–Guérin (BCG) vaccinations in reducing childhood leukemia incidence (LI). Rosenthal is credited for making the remarkable observation of reduced LI among <6-year-old children (born 1964-1969, Chicago) who had neonatal BCG inoculations (2, 9). It was suggested that in the settings that lacked sufficient and timely exposure to common microbes/pathogens, the early neonatal vaccination (<1-year) could be providing the required stimulus to children’s developing immune system for its ‘normal’ maturation (5, 8–10). Other modifiers (*e.g*., genetic, environmental, breast-feeding, diet, gut microbiota, *in-utero* modulation/reprogramming, etc.) have been also suggested to play a role in childhood LI (8–11).

Despite several early studies suggesting a protective role of BCG vaccination on early childhood LI, the outcomes from clinical trials and various long-term ecological studies with observation periods stretching over 5-60 years have been largely inconclusive due to conflicting outcomes, casting doubts over its protective or preventive potential (6, 9, 10, 12). The reasons behind variable outcomes in different studies have remained an enigma. It may be pertinent here to remind the scientific community that, a) LI rate is highest in 0-4Y-olds [<5-year-olds; acute lymphoblastic leukemia (ALL) subtype comprises up to 75% of total LI – that mostly peaks between 2-5-years of age in developed societies/populations (8)] and it registers a sharp decrease to about 1/3 to 1/4 incidences in 5-14Y-olds (>5 to <15-Year-olds) then reaches almost background levels for the majority (https://gco.iarc.fr/today/home), on supposed immune maturation; b) development of BCG-conferred “trained immunity” requires “boosting” and it seldom lasts a few years in the absence of a rechallenge (13, 14). Remarkably, the presence of tuberculin immunoreactivity (a trained immunity measure) in children had been found negatively correlated with LI (7), and still today some of the lowest LI reporting countries among 0-4Y-olds (<5Y-olds) are the BCG vaccinating not the non-vaccinating countries. As the majority of early childhood leukemia in <5-year-olds is supposed to arise from inappropriate immune stimulation and maturation, we propose that the extrapolative conclusions drawn about the impact of BCG vaccination on early childhood leukemia from the studies performed on higher age groups or from longer observation/follow-up periods, where cause(s) other than inappropriate immune maturation could be a primary reason (6, 8, 9), should be disregarded (10, 12). Seemingly, the lack of clarity about appropriate comparable control groups in many studies stemmed from confusion over the longevity of BCG-conferred trained immunity (TI) and its measurement (13, 14) and non-differentiation of possible vaccine-preventable and non-preventable causes (8, 9). Additionally, omission of differential exposure to microbes/pathogens/potential etiological agents in the compared populations as a variable, control for the actual TI status of children and interacting mother/parent/population that may be important for the transmission of supposed etiological agent(s) as well as modulation and timely maturation of children’s TI (8, 11), the inherent differences in BCG vaccines (15), *etc.* could also be responsible for some of the existing conundrum.

The environmental stimulus had been postulated previously as a likely variable for the observed differential protective impact of BCG vaccination on childhood LI in some studies (6). However, the environmental stimulus as a variable impacting early childhood LI in BCG-vaccinated children has largely remained unexplored and uncorroborated for the last 40 years. We surmise that environmental mycobacteria (*Mycobacterium* spp.) exposure could be an important driver of immune maturation and possibly causatively correlated with reduced early childhood LI. We hypothesized that if supposed TI conferred by childhood BCG vaccination and/or exposure to environmental mycobacteria played a protective role in early childhood LI, the vaccination coverage among countries and the prevailing tuberculin immunoreactivity [tuberculin skin test (TST) positive]—a measure of existing TI from previous exposure to mycobacteria (BCG and environmental) in the population (that also reflects the probability of children’s developing immune system getting primed, stimulated, trained, and boosted by natural exposure)—would be negatively correlated with early childhood LI. The countries of WHO’s European region (ER) with more similar genetic makeup as compared to the rest of the world but variable childhood LI in 2020 (**Table 1**; GLOBOCAN 2020; https://gco.iarc.fr/today/home) and known graded TST positivity [estimates also called “Latent Tuberculosis Infection (LTBI)” for purely technical reasons as mandated by the WHO for the purpose of tuberculosis surveillance and management, as a very small minority of them could develop tuberculosis in their lifetimes (14)] offer an ideal setting to evaluate the role of BCG vaccination on early childhood LI. Almost half of the ER countries have neonatal BCG vaccination (BCG) in place, while the other half of the ER countries do not have such a policy (No BCG) (**Table 1**; http://www.bcgatlas.org/index.php). Additionally, the coverage of BCG as well as other common childhood vaccines (*i.e.*, DTP3, MCV2, PCV3) in 1-year-olds that have also been suggested previously to likely have a role in stimulating protective TI (10, 12), are diverse in these countries (**Table 1**; WHO-UNICEF Immunization Coverage Estimates 2021 revision; https://data.unicef.org/topic/child-health/immunization/), making them suited for such analysis. For methodology details, refer to **Supplementary “Materials and Methods”**.

**Table 1:** Early Childhood (<5Y or 0-4Y-olds) Leukemia Incidence in European Region: ‘Trained-Immunity’ Prevalence (TST Positivity), childhood vaccination coverage and correlation analysis.

Country			Average Childhood Vaccination Coverage (%) (1Y-old) 2016-2020^a^				TST Positive Pop. (%)^b^	Leukemia Incidence: 0-4Y-old (2020)^c^
			BCG	DTP3	MCV2	PCV3		
***Hungary***			99	99	99	98.8	13.026	7.6
***Armenia***			99	92.6	96	92.8	15.555	2.9
***Albania***			98.8	98.6	96.4	96.6	14.242	8.4
***Uzbekistan***			98.4	97.4	99	77.4	16.559	3.4
***Tajikistan***			98.2	96.4	97.2	0	19.640	0.96
***Croatia***			98.2	93.2	94.4	0	11.817	8.7
***Turkmenistan***			98	98.6	99	4.6	17.286	3.8
***Serbia***			97.8	94.4	89.2	49.2	14.014	8.8
***Belarus***			97.6	97.4	98	0	13.754	7.3
***Lithuania***			96.8	92.6	92	82.2	13.358	9.6
***Georgia***			96.6	91.6	89	80.4	16.853	3
***Kyrgyzstan***			96.6	92.8	96.2	73.2	17.002	2.5
***Azerbaijan***			96.4	92	93.4	92.4	16.711	3.8
***Latvia***			96.4	98	92.4	86	13.349	5.3
***Bulgaria***			96.2	92	89.2	88.4	14.380	5.8
***Bosnia and Herzegovina***			95.8	74.4	77.2	0	15.745	3.8
***Republic of Moldova***			95.6	89.4	94.2	78.6	17.047	6.9
***Turkey***			95.4	97.8	87.8	96.6	12.459	8.9
***Russian Federation***			95.4	97	96.8	71.8	15.648	7
***North Macedonia***			95	90.8	89.2	6	14.715	7.2
***Romania***			94	86.4	76.6	34.6	13.794	4.8
***Kazakhstan***			93	92.8	97	93.6	15.236	2
***Estonia***			92.8	92	89.6	0	12.030	10.1
***Poland***			92	94.8	93	36.4	12.880	7.2
***Ukraine***			85.2	59.8	75.8	0	15.954	6.9
***Montenegro***			81.6	86.2	82.4	0	12.953	5.4
***Sweden***			25.6	97.4	93.8	97	10.071	7.8
***Ireland***			3.6	94.4	0	88.8	8.145	7.3
***Slovakia***			0	96.4	97.4	96	12.703	5.3
***Denmark***			0	96.6	88.6	96	8.805	7.1
***Greece***			0	99	83	96	9.362	8.1
***Luxembourg***			0	99	88.4	95.6	8.175	3
***Norway***			0	96.4	93	94.6	8.458	8.3
***Israel***			0	97.2	96.2	94.2	8.966	5
***Belgium***			0	97.6	85	94	8.751	9.3
***The Netherlands***			0	94	89.8	93.2	8.285	7.2
***Portugal***			0	98.6	95.4	92.6	10.328	7
***France***			0	96	83	92	8.859	6.1
***United Kingdom***			0	93.6	87.8	91.6	9.553	7.6
***Italy***			0	94.8	86.2	90.8	12.872	7.6
***Iceland***			0	91.2	94.4	90.6	7.674	4.9
***Finland***			0	90.6	91.2	87	8.284	8.3
***Switzerland***			0	96	90	83.6	8.417	8.6
***Germany***			0	91	93	82	9.202	7.9
***Cyprus***			0	97	88	81	9.062	7.7
***Spain***			0	95.4	94	74.2	6.064	6.5
***Slovenia***			0	94.2	93.2	59.8	11.062	6.9
***Malta***			0	97.6	91.6	0	9.455	4.6
***Czechia***			0	96.6	88.8	0	11.405	5
***Austria***			0	86.4	86.2	0	8.689	8
**AVERAGE**			***50.18***	***93.5***	***89.16***	***64.20***	***12.17***	***6.34***
**STD. DEV.**			***47.74***	***6.55***	***14.06***	***38.46***	***3.31***	***2.17***
PEARSON CORRELATION ANALYSIS								
**Correlation: *Leukemia Incidence per 100k (0-4Y-olds) vs Protective Variable* (Vaccination coverage/TST positivity (TI) prevalence**		All countries:						
		***r(50)*:**	**- 0.2371**	**0.0620**	**-0.1285**	**0.0943**	**- 0.4772**	
		*p-*value:	n.s. (0.0973)	n.s. 0.6688	n.s.(0.3736)	n.s.(0.5148)	<0.0005	
		**BCG** (>90% coverage)						
		***r(24)*:**	**-0.1158**	**0.1798**	**- 0.0992**	**- 0.0663**	**- 0.7868**	
		*p-*value:	n.s.(0.5901)	n.s.(0.4005)	n.s.(0.6448)	n.s.(0.7583)	<0.0000	
		**No BCG** (0% coverage)						
		***r(22)*:**	—	**-0.2694**	**- 0.3245**	**0.2006**	**- 0.0727**	
		*p-*value:	—	n.s.(0.2254)	n.s.(0.1406)	n.s.(0.3707)	n.s.(0.7479)	

The values are rounded off to the indicated decimal places. n.s.: p-value >0.05.

Only the Trained-Immunity correlate (TST positivity) was found strongly correlated with reduced Leukemia incidence in early childhood which also substantially increased for BCG vaccinated (blue box).

a Average vaccination coverage estimates for 1Y-olds born during 2016-2020 (BCG: One dose of Bacillus Calmette–Guérin; DTP3: three doses of the combined diphtheria, tetanus toxoid, and pertussis; MCV2: Measles-containing-vaccine second-dose; PCV3: three doses of pneumococcal conjugate vaccine.) as per WUENIC. WHO UNICEF Immunization Coverage Estimates 2021 revision (completed 15 July 2022). [Available from https://data.unicef.org/topic/child-health/immunization/; Last accessed 14 Jan 2023].

b TST positivity (LTBI) prevalence in respective populations is from the Global Burden of Disease Collaborative Network. Global Burden of Disease Study 2017 (GBD 2017) Results. Seattle, United States: Institute for Health Metrics and Evaluation (IHME), 2018. [Available from http://ghdx.healthdata.org/gbd-results-tool; search term combination “Prevalence—Latent Tuberculosis Infection—Sex: Both—Age: All Ages (Percent)”].

c The estimates of pediatric age-standardized leukemia incidence rates (per 100,000) in children 0-4Y-old (2020) are from the GLOBOCAN 2020 study, International Agency for Research on Cancer, World Health Organization. The sources and methodology employed for the global cancer incidence estimates for GLOBOCAN 2020 are described at the Global Cancer Observatory (GCO) website gco.iarc.fr [Available at https://gco.iarc.fr/today/home; Last accessed 14 Jan 2023].

## Vaccinations and early childhood leukemia incidences

An examination of the epidemiological cancer incidence data of ER countries (n=50) for the year 2020 (GLOBOCAN 2020 study, https://gco.iarc.fr/today/home) that potentially had comparable confounding variables, for the covariation of estimated LI in 0-4Y-olds (most affected age group; https://gco.iarc.fr/today) with the average coverage of early childhood vaccines for the indicated cohort (*i.e.*, vaccination of 1-year-olds during previous relevant years, *i.e.*, 2016-2020 from the WHO-UNICEF Immunization Coverage Estimates 2021 revision (https://data.unicef.org/topic/child-health/immunization/) presents an interesting scenario (**Table 1**). Neither the coverage of BCG nor other test vaccines (*i.e.,* DTP3, MCV2, and PCV3) was found to be significantly correlated with LI rate (**Table 1**). However, the negative correlation seemed strongest for BCG [*r*(50): -0.2371, p-value: 0.0973] as had been observed previously in reported meta-analyses (10, 12). The association was much weaker for MCV2, PCV3, and DTP3 vaccinations.

## Tuberculin immunoreactivity prevalence in BCG vaccinating countries negatively associated with early childhood leukemia incidence

**Figure 1:**
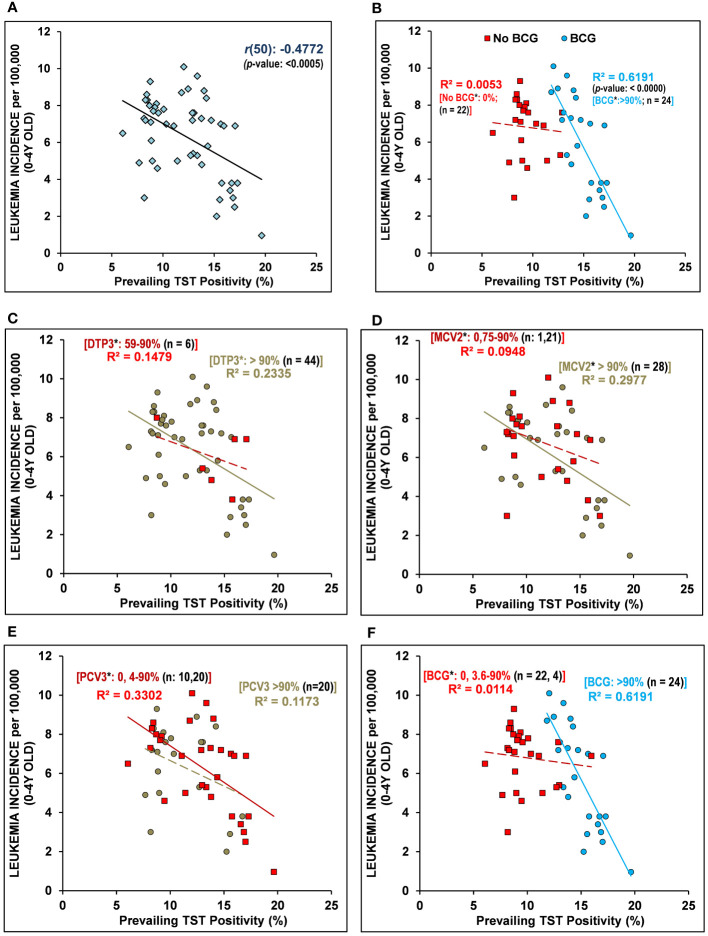
Early childhood leukemia incidence (LI) in 2020 negatively correlated with “Trained Immunity” prevalence in BCG-vaccinating countries. **(A)** The prevalence of tuberculin sensitivity test (TST) positivity – a measure of BCG vaccination or environmental *Mycobacterium* spp. exposure conferred “trained immunity” in children/individuals without active tuberculosis, is negatively correlated with leukemia incidence (LI) in 0-4Y-olds (<5Y-olds) [*r*(50): - 0.4772, *p-*value: <0.0005]. **(B)** The early childhood (0-4Y-olds) LI rates are found to be more strongly negatively correlated with the populations’ TI correlate (TST positivity) in BCG-vaccinating countries (BCG) with >90% BCG vaccination coverage in 1-Year-olds, unlike that observed for the non-vaccinating countries (No-BCG). The LI in 0-4Y-old children population that had been differentially vaccinated with DTP3 **(C)**, MCV2 **(D)**, PCV3 **(E)**, and BCG **(F)** also negatively correlated with the TI correlate (TST positivity) of the population. Note: The negative association of populations’ TI correlate (TST positivity) with the early childhood LI with regard to coverage of other childhood vaccinations (*e.g.,* DTP3, MCV2, PCV3) is substantially weaker than that for BCG (R^2^-values ranged from 0.1173 to 0.2997 for DTP3, MCV2, PCV3, as compared to 0.6191 for BCG). The inclusion of partially BCG-vaccinated countries in the non-vaccinated or vaccinated group did not affect the overall relationship/correlation. [n = number of countries; *average vaccination coverage (%) in 1-Year-olds for 2016–2020; R^2^: coefficient of determination; regression line is solid for p-values <0.05, while it is dashed/broken for *p*-values >0.05].

Next, discounting the vaccinations or their coverage, when the prevalence of tuberculin immunoreactivity (TST positivity/LTBI prevalence) from the Global Burden of Disease Study 2017 (GBD 2017), Institute for Health Metrics and Evaluation (IHME), 2018 (16), a measure of the persistence of cell-mediated trained-immunity resulting from exposure of the populations to *Mycobacterium* spp. (environmental or BCG vaccine) (14) is assessed, it is found to be significantly negatively correlated with early childhood (*i.e.,* 0-4Y-olds) LI rates [Pearson’s correlation coefficient, *r(50)*: -0.4772, p-value:<0.0005, See **Table 1** and **Figure 1A**]. The surprisingly strong negative correlation observed led us to postulate that if the TI prevalence is playing a role in augmenting supposed protective immune maturation in children that is causing reduced early childhood LI, it would be more highly correlated in countries with neonatal BCG vaccination in place (>90% coverage) than those without it (no-BCG countries). Indeed, for BCG-vaccinating countries (n=24; coverage >90%), the negative correlation of early childhood LI rate with the prevailing TI of population substantially improved [*r(24)*: -0.7868, p-value: <0.0000], whereas for no-BCG countries (n=22) it became uncorrelated (**Table 1** and **Figure 1B**). The negative association of TI prevalence with the childhood LI rate in these countries was found to be strongest for BCG while much weaker for other childhood vaccinations, as reflected in their coefficients of determination or R^2^ values [BCG(n=24): R^2 ^= 0.6191; DTP3(n=44): R^2 ^= 0.2335; MCV2(n=28): R^2 ^= 0.2977; PCV3(n=20): R^2 ^= 0.1173; **Figures 1C–F**], supporting a protective role for neonatal BCG vaccination. However, the existence of an almost similar mid-to-high LI rate per 100,000 (4-8 and >8, respectively) in low TST-positive (<15%) countries both with or without BCG coverage (*i.e.,* BCG and no-BCG countries), together with clustering of most of the low LI rate (<4) countries (9/10) in BCG-vaccinating populations with high TST positivity (>15-20%) potentially suggests an essential role of further boosting by environmental *Mycobacterium* spp. exposure in reducing LI in children primed with neonatal BCG vaccination. The observations are in conformity with the statistical likelihood of getting boosted by exposure to environmental mycobacteria to increase in high TST-positive populations.

## Discussion

There may be several competing possibilities, not necessarily exclusive of each other, which could be contributing to the overall strong negative correlation observed between populations’ TI and the early childhood LI in BCG countries. Firstly, a potentially direct causative relationship arising from a higher chance of exposure to immune-boosting/maturing events in the form of environmental mycobacteria in the high TST-positive populations could have a curative impact something akin to that suggested for the curing of certain immune disorders on tuberculin exposure even in TST positive or primed subjects (9). Secondly, qualitative differences in the modulation of children’s immune systems by their interaction with their mother, caregiver, or other immediate interacting individuals (8, 9, 11) may be playing a protective role. Thirdly, a decrease in unknown risk factors or the transmission of potential etiological agents to young children by a high TI population could be suspected. Fourthly, a favorable endocrine or *in-utero* programming by TST-positive mothers (*i.e*., exposed to mycobacterial antigens) also remains a possibility (8, 9, 11). Though all scenarios are equally possible and we do not prefer one over another, these countries with different LI rates in 0-4Y-olds (<5Y-olds) and their decrease in 5-14Y-olds [https://gco.iarc.fr/today] offer the opportunity of evaluating all these possibilities and help establish their role both qualitatively as well as quantitatively, if any. The findings would have the potential to significantly improve our understanding of “normal” immune system development during early childhood and the key players involved. More specifically, the role of neonatal BCG vaccination and exposure to microbes and common pathogens in the early-life immune training and maturation process, along with their impact on early childhood leukemia incidence, would get identified and established.

The observations presented here are not in conflict with previous observations and the hypotheses proposed concerning the potential mechanism of action, whether it be the elimination of embryonic cancer precursors or an environmental stimulus necessary for immune maturation needed for suppression or elimination of certain T-cell subsets during normal immune system development in children lacking other natural stimuli (5, 8, 9, 11); rather, they suggest the insufficiency of a single dose of BCG in bringing about the required changes in the immune system of 0-4Y-olds in the absence of rechallenge in low TST positive populations, as would have been assumed from the major extant hypotheses (6, 9, 10). Additionally, the extension of the observation presented here (*i.e.*, a 5-to 10-fold decrease in LI rate) may suggest that the possible proportion of preventable leukemia with immunological underpinnings being much more than previously estimated (8, 11) and it may not be just limited to ALL alone.

## Conclusion

The potential benefit of BCG vaccination in reducing early childhood LI in low-TI populations, a majority of which supposedly arise from a lack of appropriate early-life immune training during immune system development, has so far remained inconclusive for lack of convincing evidence. However, well-controlled clinical trials, preferably conducted in low TI populations (<10-15%) with high LI incidence in 0-4Y-olds and carefully controlling for the TI of child and mother (parents/caregiver), with and without boosters, could help resolve the controversy and provide a thorough understanding of the observations made so far, including ours that are presented in the current article. It may pave the way for again securing benefits from this over 100-year-old vaccine for early childhood LI reduction. The low-TI countries with high LI rates in 0-4Y-olds (per 100,000), like Estonia (10.1), Lithuania (9.6), Belgium (9.3), Turkey (8.9), Serbia (8.8), Croatia (8.7), Switzerland (8.6), etc., are going to gain the most if such controlled clinical trials prove to be of clinical benefit from revised vaccination regimens.

## Author contributions

SS conceived the idea, designed the research, analyzed the data, and wrote the paper. RS and DK wrote the paper. All authors contributed to the article and approved the submitted version.
