# Supplementary material for: “Trained Immunity” from Mycobacterium spp. exposure (BCG vaccination and environmental) may have an impact on the incidence of early childhood leukemia
Source: Front Immunol. 2023 May 24;14:1193859. doi: 10.3389/fimmu.2023.1193859 (PMC10244714; doi:10.3389/fimmu.2023.1193859)
Supplement: Supplementary file 1 [file DataSheet_1.docx]

***SUPPLEMENTARY MATERIAL FOR***

**“Trained Immunity” from *Mycobacterium* spp. exposure (BCG vaccination and environmental) may have an impact on the incidence of early childhood leukemia**

**Samer Singh, PhD ^1,#^; Dhiraj Kishore, MD, FICP^2^; Rakesh K. Singh, PhD^3^**

^1^Centre of Experimental Medicine & Surgery, Institute of Medical Sciences, Banaras Hindu University, Varanasi – 221005, India

^2^Department of General Medicine, Institute of Medical Sciences, Banaras Hindu University, Varanasi – 221005, India

^3^Department of Biochemistry, Institute of Science, Banaras Hindu University, Varanasi – 221005, India

*^#^* ***Corresponding Author’s E-mail*:** [samer.singh10@bhu.ac.in](mailto:samer.singh10@bhu.ac.in)

**The File contains**

1. Materials and Methods (page 2)
2. **MATERIAL AND METHODS:**

The estimates of age-standardized leukemia incidence (LI) rate per 100,000 in the indicated age group (0-4Y old or <5Y old) for 2020 are from the GLOBOCAN 2020 study (https://gco.iarc.fr/today/home). The average coverage of different childhood vaccines for the indicated-year cohort of children, i.e., 2020, is from the WHO-UNICEF Immunization Coverage Estimates 2021 revision (https://data.unicef.org/topic/child-health/immunization/). The TST/LTBI estimates for the European region (ER) countries are from the Global Burden of Disease Study 2017 (GBD 2017), Institute for Health Metrics and Evaluation (IHME), 2018 (http://ghdx.healthdata.org/gbd-results-tool) [16]. Basic statistical analysis, including correlation and regression analysis, was performed in Excel 2019 without any data transformation. P-values <0.05 are considered significant.
